# Long Noncoding RNA LIFR-AS1: A New Player in Human Cancers

**DOI:** 10.1155/2022/1590815

**Published:** 2022-01-13

**Authors:** Zhiqun Bai, Xuemei Wang, Zhen Zhang

**Affiliations:** The First Affiliated Hospital of China Medical University, Department of Ultrasonic Diagnosis, No. 155 Nanjing North Street, Heping District, Shenyang, Liaoning 110001, China

## Abstract

Emerging evidence has indicated that aberrantly expressed long noncoding RNAs (lncRNAs) play a vital role in various biological processes associated with tumorigenesis. Leukemia inhibitory factor receptor antisense RNA1 (LIFR-AS1) is a recently identified lncRNA transcribed in an antisense manner from the *LIFR* gene located on human chromosome 5p13.1. LIFR-AS1 regulates tumor proliferation, migration, invasion, apoptosis, and drug resistance through different mechanisms. Its expression level is related to the clinicopathological characteristics of tumors and plays a key role in tumor occurrence and development. In this review, we summarize the role of LIFR-AS1 in the development and progression of different cancers and highlight the potential for LIFR-AS1 to serve as a biomarker and therapeutic target for a variety of human cancers.

## 1. Introduction

Cancer is one of the leading causes of death worldwide, and its morbidity and mortality rate is increasing year by year. According to statistics, China's annual death toll of more than 4 million; so, tumor has been a threat to human health [1–4]. With the completion of the human genome-wide sequencing project, the study found that RNA with coding protein function accounted for only 2% of the total number of genes, 98% of which were noncoding RNA of uncoded proteins, of which long-chain noncoding RNA (long non-coding RNA, lncRNA) is one of the important members of noncoding RNA [5, 6].

LncRNAs are defined as noncoding RNAs longer than 200 nucleotides [7]. Compared with protein-coding mRNAs, lncRNAs display substantially lower abundance but stronger cell and tissue specificity [8]. The dysregulated lncRNA expression is highly related to the occurrence, development, and drug resistance of malignant tumors [9–13] and has been the subject of intense research focus over recent years [14]. Although the specific mechanisms underlying how lncRNAs regulate gene expression remain incompletely understood, it is known that they play an important regulatory role at all levels of gene expression, including transcription, translation, and posttranslational regulation [15–17]. LncRNAs are known to exert their regulatory functions by interacting with various biomolecules, including DNA, RNA, and proteins [14, 18], but much remains to be learned about the mechanisms underlying these effects.

**Figure 1:**
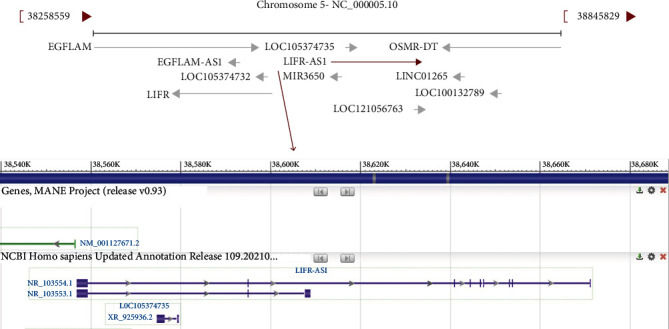
Schematic diagram of the generation of lncRNA LIFR-AS1.

**Figure 2:**
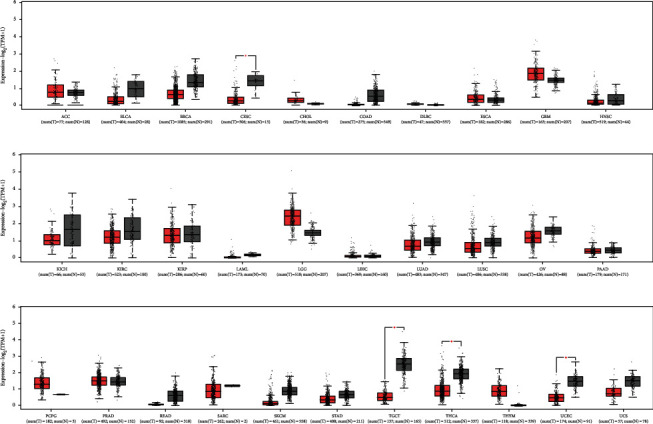
The relative expression level of LIFR-AS1 in pan-cancer and adjacent normal tissues was determined by GEPIA2 analysis based on the TCGA database. ^∗^ | Log2FC | >1, *P* < 0.01.

**Figure 3:**
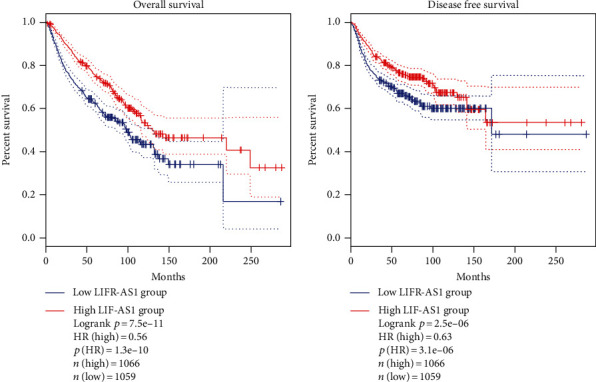
Kaplan–Meier curves for the association of LIFR-AS1 with overall survival and disease-free survival. Differentially expressed genes were ranked by the median of expression and then scored for each colorectal cancer patient according to a high or low level of expression (horizontal axis: overall survival time; vertical axis: survival function).

LIFR-AS1 is a recently identified cancer-related lncRNA transcribed in an antisense manner from the *LIFR* gene located on human chromosome 5p13.1. A search of the NCBI database (https://www.ncbi.nlm.nih.gov/) revealed that this locus can be transcribed into two transcripts, namely, NR_103553.1 and NR_103554.1 (Figure 1). To evaluate the expression of LIFR-AS1 in different types of cancer, we analyzed its expression levels using GEPIA2 (http://gepia2.cancer-pku.cn/#index), a tool based on The Cancer Genome Atlas (TCGA) database. Our investigation revealed that LIFR-AS1 was significantly downregulated in many cancers (Figure 2), and we also evaluated the prognostic value of the LIFR-AS1 expression in different cancers through GEPIA2. As shown in Figure 3, the high LIFR-AS1 expression was correlated with worse overall survival (OS) and disease-free survival (DFS). These results suggested that LIFR-AS1 may serve as a prognostic indicator in different cancers.

Extensive data have also shown that LIFR-AS1 is upregulated and plays an oncogenic role in many tumor types via different downstream targets. Herein, we review the current evidence on the abnormal expression, mechanisms of action (Table 1), and clinical significance of LIFR-AS1 (Table 2) and summarize the roles of LIFR-AS1 in multiple cancers to better understand its regulatory mechanisms.

## 2. The Function of LIFR-AS1 in Cancer and the Underlying Molecular Mechanisms

### 2.1. LIFR-AS1 in Glioma

LIFR-AS1 has been reported to be downregulated in glioma tissues and cell lines [19]. Additionally, its expression was significantly lower in grade III–IV glioma than in grade I–II disease, suggesting that LIFR-AS1 levels might reflect the grade of glioma. Meanwhile, Ding et al. also showed that the expression of LIFR-AS1 in NHA cells was significantly higher than that in U251 and A172 cells. The authors demonstrated that, in vitro, LIFR-AS1 could inhibit the proliferation, migration, and invasion of glioma cells, as well as promote their apoptosis. These results suggest that LIFR-AS1 exerts cancer suppressive effects in gliomas. Using bioinformatic and luciferase reporter assays, they demonstrated that LIFR-AS1 serves as a sponge for miR-4262, thereby competitively regulating p-NF-*κ*B p65 and I*κ*B*α* expression. In addition, they found that the LIFR-AS1 overexpression could reduce the chemoresistance of glioma cells to temozolomide (TMZ). These observations indicated that the LIFR-AS1/miR-4262/NF-*κ*B axis may play a suppressive role in glioma progression.

### 2.2. LIFR-AS1 in Thyroid Cancer

LIFR-AS1 was also found to be significantly upregulated in thyroid cancer tissues and cell lines, and its silencing reduced the viability and inhibited the proliferation of human thyroid cancer cells by inducing G2/M cell cycle arrest, possibly through the suppression of cyclin B1 and CDK1. Li et al. [20] demonstrated that the invasive and migratory abilities of thyroid cancer cells were markedly reduced under LIFR-AS1 transcriptional knockdown, which was also associated with the downregulation of the MMP-2 and MMP-9 expression. However, the precise molecular mechanisms underlying these effects were not explored. Combined, these results imply that LIFR-AS1 may promote the progression of thyroid cancer.

### 2.3. LIFR-AS1 in Breast Cancer

In one study, Xu et al. [21] first analyzed LIFR-AS1 expression data downloaded from the TCGA database and found that the LIFR-AS1 expression was significantly downregulated in breast cancer tissue when compared with that in normal tissue. qRT-PCR and in situ hybridization analyses of the LIFR-AS1 expression in clinical breast cancer samples further showed that the LIFR-AS1 expression was lower in cancerous tissues than in matched, noncancerous tissues and was also lower in breast cancer cells than in normal human mammary epithelial cells. Moreover, functional studies illustrated that LIFR-AS1 exerts inhibitory effects on breast cancer cell proliferation, colony formation, migration, and invasion in vitro. The expression of Sufu and miR-197-3p was inversely correlated with that of LIFR-AS1, and LIFR-AS1 was shown to act as a sponge for miR-197-3p in breast cancer cells. In addition, LIFR-AS1 knockdown was observed to promote tumor growth in vivo. These findings indicate that LIFR-AS1 may function as a tumor suppressor in breast cancer.

Crosstalk between dysregulated pathways occurs widely in human cancers and usually leads to insensitivity to cancer treatments. To systematically identify how lncRNAs participate in the regulation of this crosstalk in breast cancer, Wang et al. [22] proposed a strategy that integrated mRNA and lncRNA expression profiles. They identified lncRNA-mediated crosstalk pathways in four breast cancer subtypes and further found that the low LIFR-AS1 expression was associated with poor survival in patients with luminal B breast cancer. This complex crosstalk can lead to the proliferation, differentiation, and apoptosis of breast cancer cells, thereby providing useful information for understanding the pathogenesis of human cancers.

### 2.4. LIFR-AS1 in Lung Cancer

The LIFR-AS1 expression was reported to be downregulated in human lung cancer tissue compared with that in normal tissue, while its overexpression suppressed the migratory and invasive capacity of lung cancer cells. Wang et al. [23] found that LIFR-AS1 acts as a sponge for miR-942-5p, thereby inhibiting the miR-942-5p-mediated repression of ZNF471 and, consequently, also the invasive and metastatic potential of non-small-cell lung cancer (NSCLC) cells. Low LIFR-AS1 expression is also associated with poor prognosis in NSCLC patients. Combined, these observations lead the authors to propose that LIFR-AS1 plays a role in cancer suppression in NSCLC and that LIFR-AS1 as a possible therapeutic target for the treatment of NSCLC.

### 2.5. LIFR-AS1 in Gastric Cancer

Wang et al. [24] observed that the LIFR-AS1 expression was significantly upregulated in gastric cancer tissue samples relative to that in adjacent normal controls. Moreover, a receiver operating characteristic (ROC) curve analysis indicated that LIFR-AS1 may be an effective marker for differentiating between normal and tumor tissue. They further provided novel insights into the clinical relevance of LIFR-AS1, determining that this lncRNA was significantly associated with tumor size, lymphatic metastasis, and more advanced TNM stage, implying that LIFR-AS1 may be positively associated with gastric cancer progression in patients. Importantly, when the authors assessed patient survival as a function of LIFR-AS1 expression, they found that individuals with higher LIFR-AS1 expression experienced poorer clinical outcomes, with shorter average OS and DFS. Taken together, these results demonstrated that LIFR-AS1 promoted the progression of gastric cancer; however, no detailed information was provided for these LIFR-AS1-related mechanisms.

Consistent with the above results, Pan and colleagues [25] demonstrated that LIFR-AS1 positively regulated the expression of COL1A2 through sponging miR-29a-3p in both gastric tumor tissues and BSG823, HS746T, 9811, BGC803, MKN28, MGC803, and BSG823 cell lines. In addition, inhibiting LIFR-AS1 expression was found to suppress the proliferation, invasion, and migration of gastric tumor cells, as well as induce their apoptosis. These observations clearly demonstrate that LIFR-AS1 plays an important role in the occurrence and development of gastric tumors and may serve as a diagnostic tool for this disease.

In contrast to the above results, the expression of LIFR-AS1 has been reported to be reduced in both gastric tumor tissue and MKN45 and AGS cell lines [26]. Moreover, bioinformatic analysis and dual-luciferase reporter assays showed that LIFR-AS1 positively regulates the MTUS1 expression by sponging miR-4698, thereby inhibiting the MEK/ERK pathway. These authors further demonstrated that upregulated LIFR-AS1 suppressed the progression of gastric cancer via suppresses a series of gastric cancer cell behaviors, such as their proliferative, migratory, and invasive abilities.

### 2.6. LIFR-AS1 in Clear Cell Kidney Cancer

To unravel the lncRNA–miRNA–mRNA regulatory network in clear cell kidney cancer (KIRC), Zhu et al. [27] undertook a comprehensive bioinformatic analysis of the RNA-seq/miRNA-seq data from 530 KIRC cases in TCGA and generated a global lncRNA-miRNA-mRNA competing endogenous RNA (ceRNA) network in KIRC. Moreover, relevant lncRNA-related survival and expression pattern analysis demonstrated that the high expression of LIFR-AS1 was positively correlation with the overall survival of patients with KIRC. These results suggest that LIFR-AS1 plays a role in cancer suppression in KIRC, while the precise biological functions of the identified lncRNAs have not been reported to date.

### 2.7. LIFR-AS1 in Colorectal Cancer

Colorectal cancer is one of the most frequently diagnosed malignant tumors globally, and the prognosis of patients with advanced disease is poor. This highlights the urgent need to identify disease-specific biomarkers and new treatment strategies to improve the diagnosis and prognosis of colorectal cancer patients [28, 29]. Liu et al. [30] reported that LIFR-AS1 was highly expressed in colorectal cancer, and that the elevated LIFR-AS1 expression was significantly associated with the proliferation and apoptosis of colorectal cancer cells. They further suggested that LIFR-AS1 was negatively correlated with the miR-29a expression and positively correlated with *TNFAIP3* mRNA levels. Considering miR-29a was negatively correlated with TNFAIP3 mRNA in CRC, Liu et al. demonstrated that LIFR-AS1 could promote the proliferation and apoptosis of colorectal cancer cells via regulating the miR-29a/TNFAIP3 pathway. These results suggest that LIFR-AS1 may exert an oncogenic role in colorectal cancer through the regulation of this pathway; however, more direct evidence is needed to confirm this possibility.

In addition, Liu et al. [30] also found that LIFR-AS1 knockdown attenuated the resistance of colorectal cancer cells to photodynamic therapy (PDT), which has been used for treating tumors since 1997. To understand the regulatory circuits involved in RNA crosstalk that might affect colorectal cancer resistance to PDT, the expression profiles of lncRNAs and miRNAs in PDT-treated and untreated HCT116 cells were compared using Clariom D microarray analysis. The results showed that LIFR-AS1 was the most upregulated lncRNA under PDT treatment.

Liang and colleagues [31] used bioinformatic analysis to predict and determine the colorectal cancer-related ceRNA network and identified differentially expressed genes based on the gene expression profiles of colorectal cancer tissues and normal tissues obtained in TCGA. These data, in combination with real-time quantitative polymerase chain reaction (qPCR) analysis, were used to detect prognosis-related lncRNAs in colorectal cancer cell lines. The authors identified a total of 81 lncRNAs, including LIFR-AS1 and further predicted that miR-182, miR-106a, miR-372, miR-144, and miR-206 formed a ceRNA network with LIFE-AS1, thereby affecting the prognosis of colon cancer. Although these results indicated that this ceRNA network plays an important role in the occurrence and development of colon cancer, the underlying mechanisms were not determined. Tan et al. [32] also constructed a ceRNA network through the TCGA and GEO databases to determine the potential microenvironmental microbiota-mediated mechanism involved in the occurrence and progression of colorectal cancer, and they further identified LIFR-AS1 as an independent prognostic factor for colorectal cancer.

### 2.8. LIFR-AS1 in Osteosarcoma

Osteosarcoma originates from mesenchymal stem cells and is the most common primary malignant tumor of the skeletal system [33, 34]. The cancer has a complex pathology, highly invasive, and metastatic and is associated with poor prognosis. Although treatment methods and drugs are constantly updated, osteosarcoma patients still develop resistance to these drugs, leading to treatment failure [35–40]. Recent studies have shown that the tumor microenvironment plays a key role in the development of tumor chemotherapy resistance, and the infiltration of immune cells, especially macrophages, is an important factor in this process [41]. Tumor-associated macrophages (TAMs), important immune cells in the tumor microenvironment, can directly or indirectly limit the antitumor activity of chemotherapeutic drugs [42, 43]. Exosomes, nanoscale vesicles secreted by cells, can transport a variety of biologically active molecules such as protein, DNA, mRNA/miRNA/lncRNA, and lipids into the tumor cells to proliferate, invade, and invade the mother cells. Meanwhile, metastasis and chemotherapy resistance play an important role [44, 45]. Zhang et al. [46] sought to clarify the role of macrophage-derived exosomal lncRNAs in the occurrence and development of osteosarcoma, as well as determine the underlying mechanisms. For this, they sought to identify dysregulated lncRNAs and miRNAs in osteosarcoma cells cocultured with macrophage-derived exosomes through high-throughput microarray analysis and found that LIFR-AS1 was highly expressed in osteosarcoma tissues and cells, in which LIFR-AS1 might play as an oncogenic role in osteosarcoma. Reducing its exosomal expression could weaken the effect of macrophage-derived exosomes on the growth of osteosarcoma cells, whereas inhibiting miR-29a reversed these effects. Additionally, the authors reported that attenuating the LIFR-AS1 expression could partially reverse the antitumor effect of miR-29a on osteosarcoma cells, and that macrophage-derived exosomes could promote the growth and metastasis of osteosarcoma. NFIA is highly expressed in osteosarcoma patients with pulmonary metastasis, and LIFR-AS1 can be promoted through the miR-29a/NFIA axis. Therefore, it can be used as a new therapeutic target for osteosarcoma treatment.

### 2.9. LIFR-AS1 in Uterine Leiomyoma

Uterine fibroids and adenomyosis are the most common benign diseases of female genitalia and can seriously affect women's quality of life and fertility [47, 48]. Aissani et al. conducted a follow-up association study across candidate chromosomal regions for uterine leiomyoma (UL; fibroids) in 916 North American premenopausal women participating in the NIEHS Uterine Fibroids Study to identify loci that affect UL size. By adjusting for confounding factors, a proportional advantage model was fitted to evaluate the relationship between 2,484 single nucleotide polymorphisms (SNPs) and the size of uterine fibroids as measured by transabdominal and transvaginal ultrasound. In the design that included the controls, several genes potentially related to UL pathogenesis were found to be associated with tumor size, especially LIFR-AS1, which showed the strongest association (Bonferroni-unadjusted *P* = 0.0006) among the genes found to be differentially expressed in UL. The results of this association study showed that LIFR-AS1 may mediate the inhibition of leukemia inhibitory factor (LIF), a cytokine with a role in embryonic uterine development; however, replication analysis is needed to confirm the relationship between LIFR-AS1 and fibroid size.

### 2.10. LIFR-AS1 in Preterm Birth

Premature delivery refers to delivery occurring before 37 weeks of gestation and is an important cause of adverse neonatal outcomes. Spontaneous preterm delivery accounts for approximately 70% of premature births, while 50% of cases of spontaneous preterm delivery are due to preterm premature rupture of membranes (PPROM) [49]. Studies have shown that mediators of inflammation such as cytokines, chemokines, and metalloproteinases are closely related to spontaneous preterm labor and PPROM. Spontaneous preterm labor is a complex disease, and SNPs in inflammatory mediator genes related to its occurrence can affect the risk of spontaneous preterm labor and PPROM [50, 51]. Studying genetic susceptibility for spontaneous preterm birth is important for revealing its pathogenesis and for early prevention and intervention. To expand the existing evidence concerning specific gene variants that are involved in the pathogenesis of preterm birth, Frey et al. [52] used multiple genetic models in a cohort of African-American women to identify SNPs associated with spontaneous preterm birth before 37 weeks of gestation. Allele-based analysis showed that genetic variants related to the *MMP2*, *MMP1*, and *LIFR-AS1*, genes that play a key role in inflammation, extracellular remodeling, and cell signaling, were associated with a higher incidence of preterm birth under 34 weeks.

### 2.11. Clinical Significance of LIFR-AS1

LncRNA dysregulation can play an important role in tumor occurrence and development through affecting DNA methylation, histone modifications, chromatin remodeling, and miRNA-regulated gene expression. Some lncRNAs can inhibit or promote the proliferative and invasive abilities of tumors, and some are related to tumor pathological type and grade.

### 2.12. Regulation of Signal Transduction Pathways

Impaired activation or inactivation of signal transduction pathways can change cellular metabolic processes, growth rates, migration, or other biological behaviors. In glioma, lncRNAs can affect the formation, proliferation, invasion, and apoptosis of tumor cells by regulating signal transduction pathways. The expression of LIFR-AS1 is reduced in gliomas, and its overexpression in glioma cells can activate the NF-*κ*B pathway, thereby inhibiting their proliferation, migration, invasion, and other biological behaviors [13]. Meanwhile, the low LIFR-AS1 expression can affect the biological behavior of breast cancer cells through the MAPK pathway, promote the growth of breast cancer tissues, and is associated with poor prognosis in breast cancer patients [15, 16]. Meanwhile, LIFR-AS1 has also been reported to be underexpressed in gastric cancer tissues and cells, and reduced LIFR-AS1 expression can enhance the proliferative, invasive, and apoptotic abilities of gastric cancer cells through the MEK/ERK pathway. These observations indicate that LIFR-AS1 likely acts as a tumor suppressor in these three cancer types.

### 2.13. The Sponge Adsorption Effect

Some lncRNAs, known as miRNA sponges (or ceRNAs), can bind to miRNAs, thereby inhibiting their binding to target mRNAs and affecting the expression of miRNA target genes. This kind of regulation has been referred to as the sponge adsorption effect [53]. For example, LIFR-AS1, miR-29a, and TNFAIP3 can form a regulatory loop that affects the growth of lung cancer cells. MiR-29a can not only directly inhibit the expression of LIFR-AS1 in colorectal cancer cells but can also inhibit LIFR-AS1 and the formation of colorectal cancer by promoting the expression of *TNFAIP3*; meanwhile, LIFR-AS1 can serve as a sponge for miR-29a, which, in turn, inhibits the *TNFAIP3* expression. These interactions promote the proliferation and inhibit the apoptosis of colon cancer cells.

### 2.14. Interaction with Proteins

LncRNAs comprise a large class of important regulatory factors that participate in a variety of biological processes, especially important landmark processes such as the tumor cell cycle, apoptosis, and immune responses. Recent studies have shown that many lncRNAs directly bind proteins and regulate their activity, while several proteins have been shown to interact with LIFR-AS1. For instance, the downregulation of LIFR-AS1 inhibits the expression of cyclin B1 and CDK1 and induces G2/M cell cycle arrest [14]. At the same time, MMP2 and MMP9 maintain cell homeostasis by suppressing cell invasion and migration.

## 3. Conclusion and Perspectives

The transcriptome includes both coding (messenger) RNA and noncoding RNA, such as lncRNA, microRNA, and rRNA [54]. With the widespread application of new technologies such as gene-chip technology and high-throughput sequencing, an increasing number of lncRNAs have been identified and assessed for their roles in cancer, as well as for their potential use as tumor biological markers. LncRNAs can function as tumor suppressors, oncogenes, and transformation-stimulating factors [55–62]. However, current lncRNA-related knowledge is only the tip of the iceberg and much remains to be discovered regarding the molecular mechanisms underlying their role in pathophysiological processes [63].

**Figure 4:**
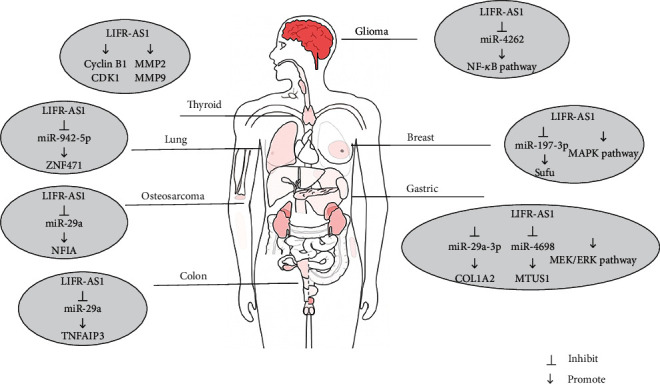
The diverse regulatory mechanisms of LIFR-AS1 in different human cancers.

The review summarized the functions of LIFR-AS1 and its receptors, which are abundantly expressed in human cancers. In addition to colon cancer and thyroid cancer, the expression of LIFR-AS1 is also related to tumor stage, tumor size, and prognosis (Table 2). Moreover, LIFR-AS1 has been associated with the chemoresistance of glioma cells to TMZ. In the diseases evaluated to date, LIFR-AS1 has been found to bind to miRNA, leading to a sponge adsorption effect, while functional experiments have shown that LIFR-AS1 affects a series of biological behaviors, including tumor proliferation, migration, and invasion. LIFR-AS1 has also been reported to regulate signal transduction pathways in glioma, breast cancer, and gastric cancer, while in thyroid cancer, LIFR-AS1 can directly affect the proliferation, migration, invasion, and cell cycle progression of thyroid cancer cells through interaction with proteins such as cyclin B1, CDK1, MMP2, and MMP9 (Figure 4).

As relatively few studies on LIFR-AS1 have been undertaken to date, further research is essential to determine its precise role in human cancers. Studies with large sample sizes are required to improve our understanding of the clinical application of LIFR-AS1 as a diagnostic/prognostic biomarker for malignant tumors. The dysregulated lncRNA expression is closely related to tumor growth, and investigating impaired lncRNA signaling is important for understanding tumor occurrence and development. LIFR-AS1 has potential as a novel prognostic indicator for a variety of cancers.

## Figures and Tables

**Table 1:** Functional characterization of LIFR-AS1 in various cancers.

Cancer type	Expression	Cell line	Role	Biological function	Related genes and protein	Reference
Glioma	Downregulated	U251, A172	Suppressor	Suppress proliferation, migration, and invasion and promote apoptosis	miR-4262 increased,	[18]
					NF-*κ*B pathway activated	
					p-NF-*κ*B p65 and I*κ*B*α* activated	
Breast cancer	Downregulated	MDA-MB-415, MCF7	Suppressor	Suppress proliferation, colony formation, migration, and invasion	miR-197-3p increased, Sufu decreased	[20]
					MAPK pathway activated	
Thyroid cancer	Upregulated	MDA-T32, MDA-T68	Oncogenic	Promote proliferation, migration, invasion,	Cyclin B1 increased, CDK1 increased	[19]
				Promote cell cycle	MMP2 increased, MMP9 increased	
Lung cancer	Downregulated	A549, H1299, PC-9, H1975	Suppressor	Suppress migration and invasion	miR-942-5p increased, ZNF471 decreased	[22]
Gastric cancer	Upregulated	BSG823, BGC803	Oncogenic	Promote proliferation, migration, and invasion and suppress apoptosis	miR-29a-3p decreased, COL1A2 increased	[23, 24]
	Downregulated	MKN45, AGS	Suppressor	Suppress proliferation, migration, and invasion	miR-4698 increased, MTUS1 decreased, MEK/ERK pathway inhibited	[25]
Colorectual cancer	Upregulated	HCT116	Oncogenic	Promote proliferation and suppress apoptosis	miR-29a decreased, TNFAIP3, miR-182 decreased, miR-106a decreased, miR-372 decreased, miR-144 decreased, miR-206 decreased	[29–31]
Osteosarcoma	Upregulated	U20S, 143B	Oncogenic	Promote proliferation and invasion and suppress apoptosis	miR-29a increased, NFIA decreased	[42]

**Table 2:** Clinical significance of LIFR-AS1 in various cancers.

Cancer type	Associated clinical features	Prognosis	Reference
Glioma	Tumor grade, chemoresistance to TMZ	Good	[18, 63]
Breast cancer	Tumor growth, poor prognosis	Good	[20]
Thyroid cancer	Tumor size	Poor	[19]
Lung cancer	TNM stage, lymph node metastasis, overall survival	Good	[22]
Gastric cancer	Progression, tumor size, lymphatic metastasis, TNM stage	/	[23–25]
Colorectual cancer	PDT treatment, prognostic factor	Poor	[29–31]
Osteosarcoma	Tumor growth, metastasis	Poor	[42]

## Data Availability

All data generated or analyzed in this study are included in the manuscript.

## Abbreviations

LncRNA: Long noncoding RNA
LIFR-AS1: Leukemia inhibitory factor receptor antisense RNA1
OS: Overall survival
DFS: Disease-free survival
TAM: Tumor-associated macrophage
SNPs: Single nucleotide polymorphisms
UL: Uterine leiomyoma
PROM: Preterm rupture of membranes
PPROM: Premature rupture of membranes
TMZ: Temozolomide
ACC: Adrenocortical carcinoma
BLCA: Bladder urothelial carcinoma
BRCA: Breast invasive carcinoma
CESC: Cervical squamous cell carcinoma and endocervical adenocarcinoma
CHOL: Cholangiocarcinoma
COAD: Colon adenocarcinoma
DLBC: Diffuse large B-cell lymphoma
ESCA: Esophageal carcinoma
GBM: Glioblastoma multiforme
HNSC: Head and neck squamous cell carcinoma
KICH: Kidney chromophobe
KIRC: Kidney renal clear cell carcinoma
KIRP: Kidney renal papillary cell carcinoma
LAML: Acute myeloid leukemia
LGG: Brain lower grade glioma
LIHC: Liver hepatocellular carcinoma
LUAD: Lung adenocarcinoma
LUSC: Lung squamous cell carcinoma
OV: Ovarian serous cystadenocarcinoma
PAAD: Pancreatic adenocarcinoma
PCPG: Pheochromocytoma and paraganglioma
PRAD: Prostate adenocarcinoma
READ: Rectum adenocarcinoma
SARC: Sarcoma
SKCM: Skin cutaneous melanoma
STAD: Stomach adenocarcinoma
TGCT: Testicular germ cell tumors
THCA: Thyroid carcinoma
THYM: Thymoma
UCEC: Uterine corpus endometrial carcinoma
UCS: Uterine carcinosarcoma.

## Additional Points

*Highlights*. (1) LIFR-AS1 is a newly discovered lncRNA. We summarize the role of LIFR-AS1 in the development and progression of different cancers for the first time. LIFR-AS1 has potential as a novel prognostic indicator for a variety of cancers.
